# Case report and brief review of literature on sonographic detection of accidentally implanted wooden foreign body causing persistent sinus

**DOI:** 10.1186/2036-7902-4-10

**Published:** 2012-05-16

**Authors:** Bhaskar Borgohain, Nitu Borgohain, Akash Handique, Parag Jyoti Gogoi

**Affiliations:** 1 Department of Orthopaedics & Trauma, North Eastern Indira Gandhi Regional Institute of Health and Medical Sciences (NEIGRIHMS), Shillong, 793018, India; 2 Department of Radiology and Imaging, North Eastern Indira Gandhi Regional Institute of Health and Medical Sciences (NEIGRIHMS), Shillong, 793018, India

**Keywords:** Sinus, Implanted foreign body, Sonography

## Abstract

Despite advances in imaging techniques, the detection of vegetative foreign bodies in soft tissues remains a difficult and sometimes even a challenging task. Clinical evaluation of such patient may present several months or even years after the initial injury and clinician may fail to elicit an antecedent skin puncture. X-ray examination will miss radiolucent foreign bodies. A 15-year-old boy presented with a draining non-healing sinus at the lateral aspect of his right thigh for 9 months. Musculoskeletal ultrasonography was ordered after ruling out chronic osteomyelitis to detect possible lesions around the thigh. High-frequency linear ultrasonic probe readily detected an elongated foreign body within the vastus lateralis muscle. A long piece of wood was confirmed at surgery. Non-healing sinus with normal finding in radiograph following old trauma should raise the suspicion of implanted radiolucent foreign body/bodies. The role of diagnostic ultrasound as a valuable screening tool for the detection of foreign body is briefly reviewed.

## Background

Despite advances in imaging techniques, the detection of retained vegetative foreign bodies remains a difficult and challenging task and may require the use of imaging modalities for better localization. Patients may present for evaluation several months or even years after the initial injury, and consequently, clinical evaluation may fail to elicit a history of antecedent skin puncture. When a history of penetrating trauma is suggested, its severity is difficult to estimate clinically [1]. Radiographs may fail to reveal the retained radiolucent foreign bodies leading to missing the diagnosis entirely [1].

## Case presentation

**Figure 1:**
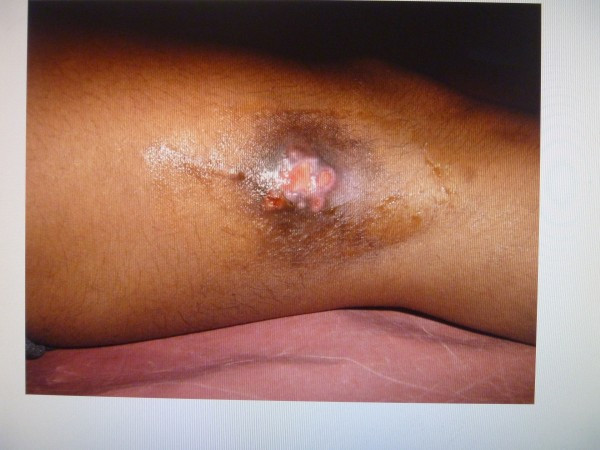
Clinical look at presentation.

A 15-year-old boy of average built presented to the orthopaedics department with a draining non-healing sinus at the lateral aspect of his right lower thigh for the last 9 months (Figure 1). Pain was minimal. The margins of the sinus were hyperpigmented and thickened with excoriation of the surrounding skin; pus was expressed out on pressure. Palpation was painful but did not reveal any mass around the sinus. Regional lymph nodes were enlarged compared to the contralateral side. Distal neurovascular status was normal. The knee joint was not swollen, and the range of motion was normal except for a mild terminal restriction of flexion due to pain. Detailed history revealed that about 9 months ago, he fell down from a tree which is approximately 15 feet in height and sustained injury from wooden branches at the right thigh. He also had transient loss of consciousness from the impact. After consciousness was regained, he noticed a small wound over the outer aspect of his right thigh. There was not much bleeding, and he considered it a minor injury. The wound, however, nearly healed. After about 3 weeks, he noticed swelling and frank pus started coming out of the wound. He sought traditional treatment and got some temporary relief. The amount of pus decreased in-between and the size also diminished, but complete healing never occurred. During the whole course, he did not develop any fever, loss of weight, cough, or chest pain. After 9 months from the day of injury, he visited our hospital.

**Figure 2:**
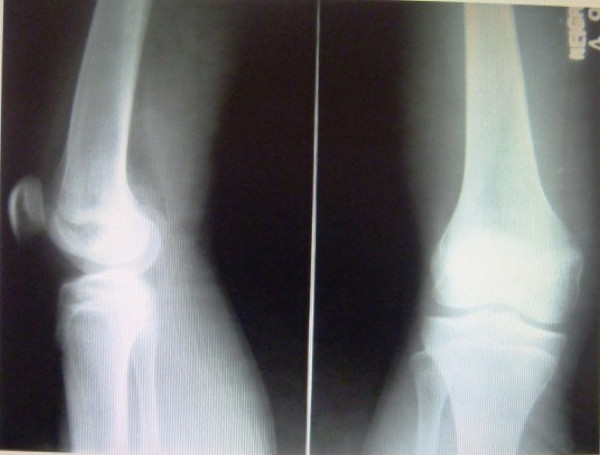
Radiograph not showing any bony lesion or sequestrum or foreign bodies.

**Figure 3:**
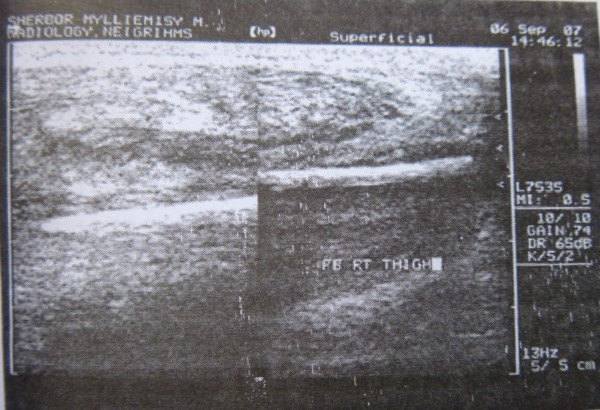
Ultrasonographic picture clearly showing the foreign body.

After admitting him for detailed evaluation, the differential diagnosis considered chronic osteomyelitis, fungal infection, tuberculosis, and implanted foreign body. Blood parameters and X-ray examination of the area came out to be normal and osteomyelitis was ruled out (Figure 2). Pus culture revealed growth of *Staphylococcus aureus*. FNAC obtained from the enlarged regional lymph node showed nonspecific findings. Implanted radiolucent foreign body was thought as a possibility for persistent discharge. Musculoskeletal ultrasonography was ordered and performed by a radiologist using a 5- to 7.5-MHz linear probe (HP image point Hx system) which readily showed a single, about 7- to 8-cm-long foreign body within the vastus lateralis muscle with surrounding edema (Figure 3).

**Figure 4:**
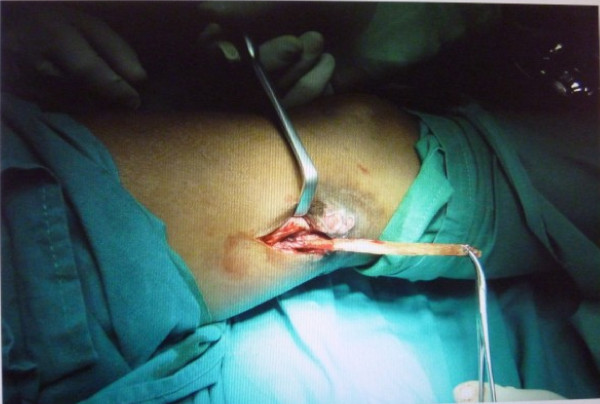
Intraoperative photograph of the removal of the wooden foreign body.

After an informed consent, the patient was taken for exploration of the wound under spinal anesthesia under a pneumatic tourniquet. The incision was made over the old scar incorporating the sinus tract and explored in a systematic manner. The vastus lateralis was approached after incising the deep fascia. Finger dissection of the muscle revealed the foreign body which was identified as a long piece of wood measuring about 1.5 × 7.5 cm (Figure 4).

Deep culture and biopsy were taken. The sinus tract was curetted and washed with hydrogen peroxide and povidone iodine solution. The incised wound was closed, leaving the sinus open. We could not use intraoperative sonography, but ordered repeat ultrasound examination to confirm complete removal of the wooden foreign body. Biopsy taken from the soft tissues surrounding the wooden foreign body demonstrated nonspecific chronic inflammation. Deep culture showed *S. aureus* again. Fungal culture could not be done. IV antibiotics were continued for 2 weeks according to culture sensitivity report. Surgical removal caused the sinus to heal uneventfully within 3 weeks.

### Discussion and brief review of literature

In various cadaveric studies [2], a sensitivity of 94% and a specificity of 99% have been demonstrated for sonography as a diagnostic modality in fresh cases. Theoretically, a combination of ultrasound and X-ray films should allow for diagnosis and localization of virtually all foreign bodies. Sonography can be used effectively to locate wooden foreign bodies as small as 2.5 mm in length [3]. Given that many foreign bodies are radiographically undetectable, the accuracy and availability of sonography make it an excellent modality for evaluation of radiolucent foreign bodies [3,4]. The imaging appearance of wooden foreign bodies is variable; however, imaging can be quite specific [1], and when taken in the appropriate clinical setting, the imaging should reliably suggest the diagnosis. Wood is highly echogenic and reveals pronounced acoustic shadowing on sonography [1]. Sonography is frequently underused but proved most useful for the evaluation of retained wooden foreign bodies [1]. Radiographs failed to reveal the retained radiolucent foreign bodies in all (12 out of 12) patients in a published report [1]. While most of the metal and glass foreign bodies can be detected by plain radiography, organic substances such as wood and vegetative materials are radiolucent, and unfortunately, these radiolucent foreign bodies are usually more prone to cause an inflammatory reaction and infection [4]. The detection can be even more difficult in cases of penetrating injuries [5] with small innocuous skin wounds. The detection of wood is especially important because it may serve as an unrecognized nidus for infection. Wood, with its porous consistency and organic nature, is an excellent medium for microorganisms, and the retained wooden foreign matter may result in cellulitis, abscess, or fistula formation [5]. The wooden foreign matter may also result in synovitis if a joint is violated or in osteomyelitis if adjacent osseous structures become involved. Radiographs have been reported to reveal a wooden foreign body in only 15% of patients and 38% of retained foreign bodies in the soft tissues were overlooked at initial examination [6]. Xeroradiography has been reported as slightly more sensitive than conventional radiography for the detection of retained wood; however, xeroradiographs show negative results in 80% of patients and are not available in most radiology departments [7]. Sonography is indeed a reliable investigation for the detection of foreign bodies in soft tissue [4]. Sonography has been well studied in the evaluation of retained foreign bodies and has been proved to be both sensitive and specific [4,8,9].

Given the markedly different acoustic impedance of wood and soft tissues, retained wooden foreign bodies are easily identified by radiologists, with the leading edge of the echogenic wood resulting in marked acoustic shadowing [10]. Retained foreign bodies in the soft tissue of the extremities that were initially overlooked and discovered later are unfortunately common [11]. Localization of embedded foreign body may prove to be very difficult. Exploration under regional or general anesthesia with application of a tourniquet (whenever possible) has been recommended to provide a better operative view [11].

Incomplete removal is a possibility, and exposure should be adequate to avoid it. Sonography can be a useful therapeutic adjuvant in the management of wooden splinter or other foreign bodies in the extremities [11]; ultrasound can confirm completeness of removal of the foreign body after surgery as well [6]. Notably, the identification of wooden foreign bodies may be exceedingly difficult on magnetic resonance imaging (MRI), especially when foreign bodies are small and there is no associated abscess or fluid collection [1]. When compared with MRI, computed tomography (CT) scan has the advantage of being less expensive, more readily available, and faster to perform. However, sonography has been proved to be effective only for superficial foreign bodies. Foreign bodies and their accompanying shadowing or reverberation may not be well visualized if they are located adjacent to the bone or deep to subcutaneous gas [2]. X-ray examinations will miss radiolucent foreign bodies. Sonography allows detection of a variety of soft-tissue foreign bodies, including wood splinters, glass, metal, and plastic, along with evaluation of their associated soft-tissue complications [11].

Ultrasound examination can readily demonstrate such ‘radiolucent’ foreign bodies. Such implanted foreign body can lead to infection. Ultrasound examination can readily demonstrate such radiolucent foreign bodies, but sonography is not commonly ordered by orthopedists and clinicians alike in traumatic conditions of the limbs unlike X-ray examination. Therefore, this participatory, real-time, cheap, and easily available diagnostic modality is often ignored as a screening tool by clinicians, and instead, there is tendency to order a high-end imaging modality like MRI or high-radiation-based modality like CT scan. This case implies that a non-healing sinus with normal bone finding in radiograph following old trauma should raise the suspicion of implanted foreign body/bodies which can be detected at bedside by office sonography instead [7].

## Conclusions

Non-healing sinus with normal bone finding in radiograph following old trauma should raise the suspicion of implanted foreign body/bodies which can be detected by sonography. Ultrasound examination can readily demonstrate radiolucent foreign bodies, but sonography is not commonly ordered by orthopedists and clinicians in traumatic conditions of the limbs as a screening tool for evaluation unlike X-ray examination. Though it is a cheap, easily available, and real-time diagnostic modality, sonography is often ignored as a screening tool in limb injuries. Instead, there is tendency among clinicians to order a high-end imaging modality like MRI or high-radiation-based modality like CT scan.

## Consent

Written informed consent was obtained from the patient for publication of this case report and any accompanying images. A copy of the written consent is available for review by the Editor-in-Chief of this journal.

## Competing interests

The authors declare that they have no competing interests.

## Authors' contributions

BB wrote the manuscript. NB and AH edited early versions of the manuscript. BB also edited the final version of the manuscript, and PJG adapted and revised the figures and figure legends. All authors read and approved the final manuscript.

## Authors' information

Dr. BB is an assistant professor and in charge and Dr. NB and Dr. PJG are former senior residents in the Department of Orthopaedics & Trauma in NEIGRIHMS. Dr. AH is an assistant professor in the Department of Radiology and Imaging in NEIGRIHMS.
